# Supplementary material for: Relationship between serum uric acid levels and periodontitis—A cross-sectional study
Source: PLoS One. 2024 Sep 27;19(9):e0310243. doi: 10.1371/journal.pone.0310243 (PMC11432880; doi:10.1371/journal.pone.0310243)

PLOS ONE

Relationship between serum uric acid levels and periodontitis

--Manuscript Draft--

**Manuscript Number:**	PONE-D-24-03627
**Article Type:**	Research Article
**Full Title:**	Relationship between serum uric acid levels and periodontitis
**Short Title:**	Relationship between serum uric acid levels and periodontitis
**Corresponding Author:**	Ping Feng<br>Guiyang Stomatological Hospital<br>Guiyang, --- Select One --- CHINA
**Keywords:**	Key words :NHANES, periodontal health, serum uric acid
**Abstract:**	Abstract<br>Objectives: Whether there is an association between serum uric acid level (sUA) and periodontitis remains unclear. The aim of this study was to investigate the association between moderate/severe periodontitis and sUA in US adults.<br>Materials and Methods: A total of 3398 participants were included in the National Health and Nutrition Examination Survey (NHANES) from 2009 to 2014. The independent variable was sUA, the dependent variable was periodontitis, and the covariates included socio-demographic variables, lifestyle variables, systemic diseases, etc. Multiple linear regression models were used to investigate the distribution of differences in covariates between different independent groups. To investigate the association between serum uric acid levels and moderate/severe periodontitis, three models were used (Model 1: unadjusted covariates; Model 2: adjusted for age, sex, and race/ethnicity; Model 3: adjusted for age, sex, race/ethnicity, education, household income/poverty ratio, smoking behavior, alcohol consumption, dental floss frequency, obesity, hypertension, diabetes, high cholesterol, hyperlipidemia, and sleep disorders)<br>Results: Among the 3398 patients, 42.5% had moderate/severe periodontitis. Multivariate logistic regression analysis showed that sUA was significantly associated with moderate/severe periodontitis (OR=1.10, 95%CI: (1.03, 1.16), P=0.0020) after adjusting for potential confounding factors. In addition, it may vary by race/ethnicity and gender. The association between sUA levels and periodontitis incidence was U-shaped in women and non-Hispanic blacks.<br>Conclusion: In conclusion, this cross-sectional study suggests that sUA levels are positively associated with the occurrence of periodontitis. However, the association between sUA levels and the occurrence of periodontitis in women and non-Hispanic blacks followed a U-shaped curve.<br>Clinical Relevance ：sUA may directly or indirectly contribute to the global burden of periodontal disease, but there is little evidence for a directed association between sUA and periodontitis. This study further supports that high sUA level is a risk factor for periodontitis and may be helpful for the control of periodontitis. It also provides new insights into whether it can be used as an indicator to assess the risk or progression of periodontitis. More studies are needed to confirm the relationship between sUA and periodontitis.
**Order of Authors:**	Jingjing Bai
	Chenying Zhou
	Ye Liu
	Ming Ding
	Zhonghua Zhang
	Zhu Chen
	Jukun Song
	Pingá Feng

Powered by Editorial Manager® and ProduXion Manager® from Aries Systems Corporation

<table>
<tr><th>Additional Information:</th><th></th></tr>
<tr><th>Question</th><th>Response</th></tr>
<tr><td>Financial Disclosure

Enter a financial disclosure statement that describes the sources of funding for the work included in this submission. Review the submission guidelines for detailed requirements. View published research articles from PLOS ONE for specific examples.

This statement is required for submission and will appear in the published article if the submission is accepted. Please make sure it is accurate.

Funded studies
Enter a statement with the following details:
• Initials of the authors who received each award
• Grant numbers awarded to each author
• The full name of each funder
• URL of each funder website
• Did the sponsors or funders play any role in the study design, data collection and analysis, decision to publish, or preparation of the manuscript?

Did you receive funding for this work?</td><td>The author(s) received no specific funding for this work.</td></tr>
<tr><td>Competing Interests

Use the instructions below to enter a competing interest statement for this submission. On behalf of all authors, disclose any competing interests that could be perceived to bias this work—acknowledging all financial support and any other relevant financial or non-financial competing interests.

This statement is required for submission and will appear in the published article if the submission is accepted. Please make sure it is accurate and that any funding sources listed in your Funding Information</td><td>The authors have declared that no competing interests exist.</td></tr>
</table>

later in the submission form are also declared in your Financial Disclosure statement.<br><br>View published research articles from *PLOS ONE* for specific examples.<br><br>**NO authors have competing interests**<br><br>Enter: *The authors have declared that no competing interests exist.*<br><br>**Authors with competing interests**<br><br>Enter competing interest details beginning with this statement:<br><br>*I have read the journal's policy and the authors of this manuscript have the following competing interests: [insert competing interests here]*<br><br>* typeset	
**Ethics Statement**<br><br>Enter an ethics statement for this submission. This statement is required if the study involved:<br><br>• Human participants<br>• Human specimens or tissue<br>• Vertebrate animals or cephalopods<br>• Vertebrate embryos or tissues<br>• Field research<br><br>Write "N/A" if the submission does not require an ethics statement.<br><br>General guidance is provided below. Consult the submission guidelines for detailed instructions. **Make sure that all information entered here is included in the Methods section of the manuscript.**	N/A

**Format for specific study types**<br><br>**Human Subject Research (involving human participants and/or tissue)**<br>• Give the name of the institutional review board or ethics committee that approved the study<br>• Include the approval number and/or a statement indicating approval of this research<br>• Indicate the form of consent obtained (written/oral) or the reason that consent was not obtained (e.g. the data were analyzed anonymously)<br><br>**Animal Research (involving vertebrate animals, embryos or tissues)**<br>• Provide the name of the Institutional Animal Care and Use Committee (IACUC) or other relevant ethics board that reviewed the study protocol, and indicate whether they approved this research or granted a formal waiver of ethical approval<br>• Include an approval number if one was obtained<br>• If the study involved *non-human primates*, add *additional details* about animal welfare and steps taken to ameliorate suffering<br>• If anesthesia, euthanasia, or any kind of animal sacrifice is part of the study, include briefly which substances and/or methods were applied<br><br>**Field Research**<br><br>Include the following details if this study involves the collection of plant, animal, or other materials from a natural setting:<br>• Field permit number<br>• Name of the institution or relevant body that granted permission	
**Data Availability**<br><br>Authors are required to make all data underlying the findings described fully available, without restriction, and from the time of publication. PLOS allows rare exceptions to address legal and ethical concerns. See the PLOS Data Policy and FAQ for detailed information.	Yes - all data are fully available without restriction

A Data Availability Statement describing where the data can be found is required at submission. Your answers to this question constitute the Data Availability Statement and **will be published in the article**, if accepted.<br><br>**Important:** Stating 'data available on request from the author' is not sufficient. If your data are only available upon request, select 'No' for the first question and explain your exceptional situation in the text box.<br><br>Do the authors confirm that all data underlying the findings described in their manuscript are fully available without restriction?	
**Describe where the data may be found in full sentences. If you are copying our sample text, replace any instances of XXX with the appropriate details.**<br><br>• If the data are **held or will be held in a public repository**, include URLs, accession numbers or DOIs. If this information will only be available after acceptance, indicate this by ticking the box below. For example: *All XXX files are available from the XXX database (accession number(s) XXX, XXX.).*<br>• If the data are all contained **within the manuscript and/or Supporting Information files**, enter the following: *All relevant data are within the manuscript and its Supporting Information files.*<br>• If neither of these applies but you are able to provide **details of access elsewhere**, with or without limitations, please do so. For example:<br><br>*Data cannot be shared publicly because of [XXX]. Data are available from the XXX Institutional Data Access / Ethics Committee (contact via XXX) for researchers who meet the criteria for access to confidential data.*<br><br>*The data underlying the results presented in the study are available from (include the name of the third party*	All relevant data are within the manuscript and its Supporting Information files

and contact information or URL).<br>• This text is appropriate if the data are owned by a third party and authors do not have permission to share the data.<br><br>* typeset	
Additional data availability information:	Tick here if your circumstances are not covered by the questions above and you need the journal's help to make your data available.

Powered by Editorial Manager® and ProduXion Manager® from Aries Systems Corporation

Click here to access/download;Manuscript;manuscript.docx

Relationship between serum uric acid levels and periodontitis

Author: Jingjing Bai 1,2 Chenying Zhou 1,2 Ye Liu 1,2 Ming Ding 1,2 Zhonghua Zhang 1,2 Zhu Chen 1,2 Ping Feng 1,2*and Jukun Song 3*

Affilication: 1Guiyang Stomatological Hospital, Guiyang, Guizhou , China;2School of Stomatology, Zunyi Medical University, Zunyi, Guizhou , China;3Stomatological Hospital Affiliated to Guizhou Medical University, Guiyang, Guizhou, China

*Corresponding Author : Feng Ping , 253 Jiefang Road, Nanming District, Guiyang, Guizhou, 550005, (China Email address: 398994584@qq.com) and Song Jukun , No. 9, Beijing Road, Yunyan District, Guiyang, Guizhou, 550002, (China Email address: songjukun@163.com)

*These authors are co-corresponding authors.

Abstract

Objectives: Whether there is an association between serum uric acid level (sUA) and periodontitis remains unclear. The aim of this study was to investigate the association between moderate/severe periodontitis and sUA in US adults.

Materials and Methods: A total of 3398 participants were included in the National Health and Nutrition Examination Survey (NHANES) from 2009 to 2014. The independent variable was sUA, the dependent variable was periodontitis, and the covariates included socio-

demographic variables, lifestyle variables, systemic diseases, etc. Multiple linear regression models were used to investigate the distribution of differences in covariates between different independent groups. To investigate the association between serum uric acid levels and moderate/severe periodontitis, three models were used (Model 1: unadjusted covariates; Model 2: adjusted for age, sex, and race/ethnicity; Model 3: adjusted for age, sex, race/ethnicity, education, household income/poverty ratio, smoking behavior, alcohol consumption, dental floss frequency, obesity, hypertension, diabetes, high cholesterol, hyperlipidemia, and sleep disorders)

Results: Among the 3398 patients, 42.5% had moderate/severe periodontitis. Multivariate logistic regression analysis showed that sUA was significantly associated with moderate/severe periodontitis (OR=1.10, 95%CI: (1.03, 1.16), P=0.0020) after adjusting for potential confounding factors. In addition, it may vary by race/ethnicity and gender. The association between sUA levels and periodontitis incidence was U-shaped in women and non-Hispanic blacks.

Conclusion: In conclusion, this cross-sectional study suggests that sUA levels are positively associated with the occurrence of periodontitis. However, the association between sUA levels and the occurrence of periodontitis in women and non-Hispanic blacks followed a U-shaped curve.

Clinical Relevance : sUA may directly or indirectly contribute to the global burden of periodontal disease, but there is little evidence for a directed association between sUA and periodontitis. This study further supports that high sUA level is a risk factor for periodontitis and may be helpful for the control of periodontitis. It also provides new insights into whether

it can be used as an indicator to assess the risk or progression of periodontitis. More studies are needed to confirm the relationship between sUA and periodontitis.

Key words :NHANES, periodontal health, serum uric acid

1. Introduction

Periodontitis is a chronic multifactorial inflammatory disease characterized by progressive destruction of dental supporting tissues, eventually leading to tooth loss [1]. Patients with severe periodontitis account for 23.6% of the global population, which is a highly prevalent disease [2]. Periodontitis not only affects oral health, but also has a two-way relationship with many chronic diseases, including diabetes, hypertension, Alzheimer's disease and coronary heart disease [3-6]. These chronic conditions impose a substantial health and economic burden. Plaque biofilm is a pathogenic factor of periodontitis, but the host immune response determines the severity of its destruction [7].The host immune system is regulated by genetic, environmental, and behavioral factors [8] , and impaired immune and metabolic responses induced by periodontal pathogens are key features in the pathogenesis of periodontitis [7].Systemic oxidative stress is an important mechanism for the progression of periodontitis [9].

Uric acid (UA), the end product of purine nucleoside catabolism, is considered to be an important free radical scavenger in the antioxidant pool [10 11].Most of the UA is excreted by the kidneys, and the remainder is broken down by the gut microbiota. UA mostly exists in the form of urate (monosodium urate in circulation and potassium/ammonium/calcium urate in urine, which activates the crystallization of uric acid in joints and extra-articular

regions)[10].Hyperuricemia occurs when UA production exceeds excretion [12]. Studies have shown that hyperuricemia is a risk factor for a variety of inflammatory diseases such as diabetes, metabolic syndrome and cardiovascular disease [12-14].Elevated UA levels within the normal range are considered to be a protective factor of bone mineral density against bone resorption, and exceeding normal levels of UA is a risk factor for osteoporosis and fractures, which is related to the imbalance between anti-oxidation and oxidative stress[15].Few studies have shown that elevated uric acid levels alter purine metabolism in oral diseases, including periodontitis and tooth resorption [16 17].

Studies have found that serum UA levels increase or sialopurine metabolism changes in patients with periodontitis [17].The level of UA in blood and saliva decreased after periodontal treatment [18] [17].Intervention using urate-lowering drugs has also shown beneficial effects in periodontitis animals [19].These findings tend to support the conclusion that there is a positive relationship between elevated serum uric acid levels and periodontitis. Serum uric acid level may aggravate periodontal tissue destruction by enhancing proinflammatory cytokines [20] , and oxidative stress [19]. Another part of scholars hold the opposite opinion, that is, high uric acid level has a protective effect on bone metabolism disorders and is independent of physiological bone metabolism [21].However, the impact of sUA on periodontitis has not been discussed as extensively in the medical literature as that of blood glucose, and we aimed to investigate the association between sUA and moderate/severe periodontitis using data from the National Health and Nutrition Examination Survey (NHANES) dataset (2009-2014). In our study, understanding the relationship between periodontitis and sUA may contribute to the prevention and control of

periodontitis.

Materials and Methods

This was a cross-sectional study using the published data set from NHANES. NHANES is a stratified, multistage, nationally representative, continuous sample survey of civilian, non-institutionalized populations in fifty states and the District of Columbia[22].NHANES data have been provided biennium since 1999. Each year, the survey assesses a nationally representative sample of approximately 5,000 people.

Study population

We used the 2009-2014 survey cycle of NHANES. Details can be found elsewhere [22].The protocol required informed consent from all participants, was approved by the ethics review board at the National Center for Health Statistics, and the data were publicly available. About NHANES laboratory/medical technical personnel method, anthropometry, laboratory procedures, a blood sample collection, storage, diagnosis, and quality standard of detailed information, see www.cdc.gov/nchs/nhanes.htm. 32,283 participants who did not have complete measurement data or whose health status did not meet the inclusion criteria were excluded. Finally, 3398 participants with completed data were analyzed in this study (FIG. 1).

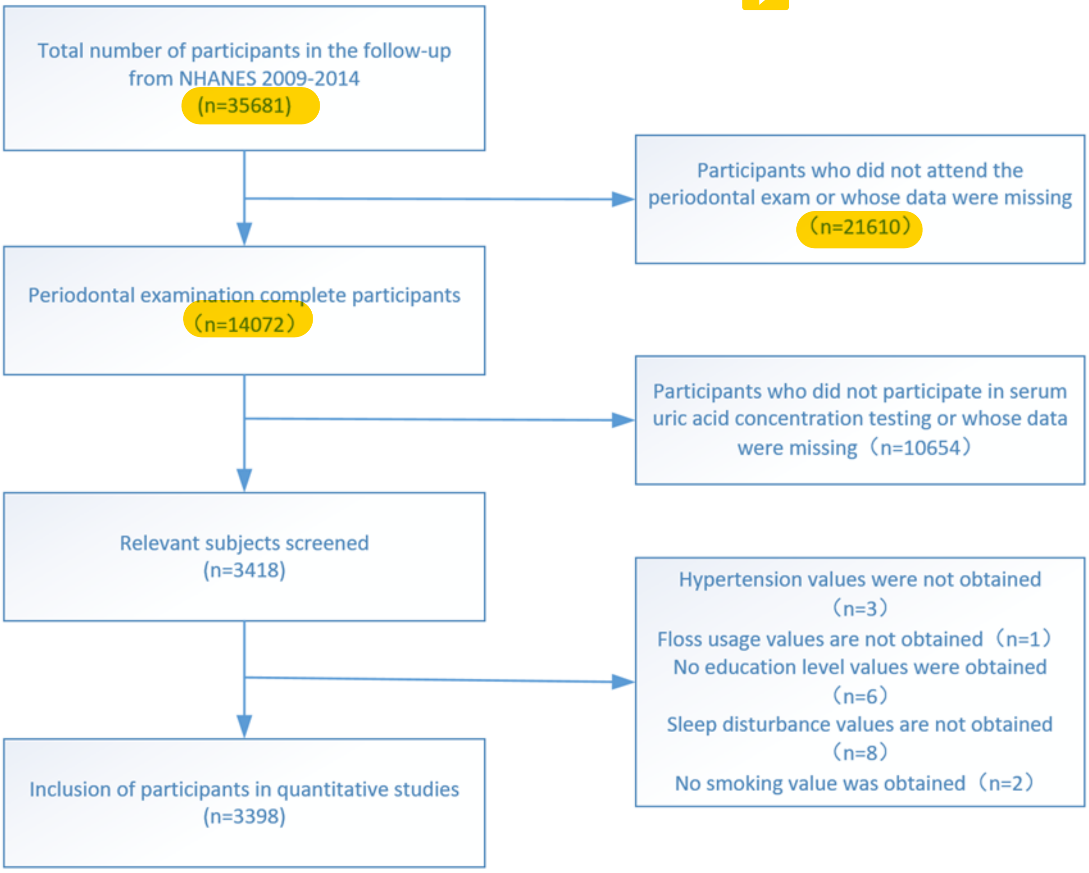

Figure 1

Sample screening flow chart.

Periodontal examination and classification

In the data set (NHANES 2009-2014), all participants were examined by a trained and calibrated dentist. Both probing pocket depth (PD) and clinical attachment loss (CAL) showed good reliability. The periodontist probed each tooth at six locations (mesial buccal, distal buccal, buccal, mesial lingual (or palate), distal lingual (or palate), and midlingual (or palate) during the periodontal examination. Here, we excluded third molars and examined only 28 teeth and 168 sites per person.

We referred to the CDC/AAP(Centers for Disease Control and Prevention and

American Academy of Periodontology) case definition of periodontitis [23] .

In order to reduce the bias caused by the high prevalence of mild periodontitis in the population, we divided patients with moderate and severe periodontitis into periodontitis categories. Patients with mild or no periodontitis were classified in the referent category[24].

Severe/moderate periodontitis was defined as follows:

≥2 proximal sites with clinical attachment loss (CAL) ≥4 mm;

Two or more proximal sites with periodontal exploration depth ≥5 mm.

Uric acid concentration

Uric acid DxC800 was used to measure the concentration of serum uric acid by timing endpoint method. Uric acid is oxidized by uricase to produce uric acid and hydrogen peroxide. Hydrogen peroxide reacts with 4-aminoantipyrine (4-AAP) and 3, 5-dichloro-2-hydroxybenzenesulfonate (DCHBS) under peroxidase catalysis to produce colored products. The system monitored the change in absorbance at 520nm at fixed time intervals. The change in absorbance is proportional to the concentration of uric acid in the sample.

Potential confounding factors

We consider the following possible confounders associated with periodontitis and uric acid. Sociodemographic variables included age, sex, body mass index (BMI), race/ethnicity, level of education, and the ratio of household income to poverty, with education categorized into three categories: less than high school, high school, and bachelor's degree or higher. BMI was divided into obese (BMI≥30kg/m2) and non-obese (BMI <

30kg/m2). The ratio of household income to poverty was divided into low (< 1.35), medium (≥1.35, < 3.0) and high (≥3.0). Lifestyle variables included smokers (never, current, quit), drinkers (at least 12 alcoholic beverages in a year), frequency of floss use (never (0 number of uses), rarely (1-2 number of uses), moderate (3-5 number of uses), and frequent (6-7 number of uses)). Physcian-diagnosed systemic diseases (hypertension, sleep disorders, high cholesterol), and diabetes mellitus (physcian-diagnosed, measured glycosylated hemoglobin >6.5%, measured fasting blood glucose ≥7mmol/L, and 2-hour glucose ≥11.1mmol/L after glucose tolerance test (OGTT)); Hyperlipidemia (measured LDL≥130mg/dl and TC≥240mg/dl).

Statistical Analysis

Statistical analyses were performed with the use of EmpowerStatsversion 4.1. Sample weights were calculated taking into account all NHANES estimates. Categorical variables were expressed as frequencies or percentages and compared using a weighted chi-square test, including sex, race, education, household income to poverty ratio, smoking history, drinking history, flossing frequency, obesity, hypertension, diabetes, high cholesterol, hyperlipidemia, and sleep disorders. Continuous variables were mean ± SD and included age, sUA.

After adjusting for potential confounders, weighted multiple logistic regression model and smooth curve fitting were used to evaluate the association between sUA and moderate/severe periodontitis. Serum uric acid was categorized according to the quartile distribution of serum uric acid concentration (Q1:1.1-4.3 mg/dL; Q2:4.4-5.2 mg/dL; Q3:5.3-

6.2 mg/dL; Q4:6.3-13.3 mg/dL), where the first quartile was designated as the reference group. Three statistical models were constructed: model 1, without covariate adjustment; Model 2, adjusted only for age and gender; Model III, adjusted for all covariates: age, sex, race, education, household income to poverty ratio, smoking history, drinking history, dental floss frequency, obesity, hypertension, diabetes, high cholesterol, hyperlipidemia, sleep disturbance, and finally, we examined gender and racial/ethnic subgroups to see if there were any sources of heterogeneity.

The weighted logistic regression model was used to calculate the difference of continuous variables. For categorical variables, a weighted chi-square test was used. A P value of less than 0.05 was considered statistically significant.

Results

Characteristics of the participants

There were no significant differences in education level and household income/poverty level ratio among the sUA groups (all $p > 0.05$).The mean age of the participants was 52.35 years, and 1865 (54.89%) were men and 1533 (45.11%) were women.Moderate or severe periodontitis was present in 42.5% of the participants. Participants in the Q4 group (individuals with high sUA concentrations) were more likely to be male, non-Hispanic white. Compared with the other groups, participants in Q4 group had higher education level, more alcohol consumption, lower frequency of dental floss, higher household income, higher proportion of poverty, and higher prevalence of obesity and cholesterol, hypertension, diabetes, hyperlipidemia, and moderate/severe periodontitis (Table 1) .

Table 1

Weighted characteristics of the study population based on quartiles of serum uric acid.

Serum uric acid categories(%)	Q1(1.1-4.3)	Q2(4.4-5.2)	Q3(5.3-6.2)	Q4(6.3-13.3)	P-value
N	777	866	852	903	
Serum uric acid (mg/dL, mean ± SD)	3.69 ± 0.52	4.82 ± 0.26	5.73 ± 0.29	7.24 ± 0.86	<0.001
Age (mean (SD))	50.33 ± 14.08	51.92 ± 15.06	53.18 ± 14.97	53.69 ± 15.09	<0.001
Sex (%)					<0.001
Male	372 (47.88%)	445 (51.39%)	499 (58.57%)	549 (60.80%)	
Female	405 (52.12%)	421 (48.61%)	353 (41.43%)	354 (39.20%)	
Race (%)					<0.001
Mexican-American	129 (16.60%)	123 (14.20%)	120 (14.08%)	89 (9.86%)	
Other Hispanics	90 (11.58%)	81 (9.35%)	65 (7.63%)	67 (7.42%)	
Non-Hispanic white	318 (40.93%)	390 (45.03%)	391 (45.89%)	388 (42.97%)	

Non-Hispanic black	123 (15.83%)	145 (16.74%)	159 (18.66%)	221 (24.47%)	
Other races	117 (15.06%)	127 (14.67%)	117 (13.73%)	138 (15.28%)	
Educational level (%)					0.278
Less than high school	154 (19.82%)	157 (18.13%)	184 (21.60%)	160 (17.72%)	
High school	161 (20.72%)	202 (23.33%)	181 (21.24%)	217 (24.03%)	
College graduate or above	462 (59.46%)	507 (58.55%)	487 (57.16%)	526 (58.25%)	
BMI categories(%)					<0.001
Non-obese(< 30 kg/m2)	559 (72.60%)	530 (61.41%)	508 (59.91%)	452 (50.11%)	
Obesity (> = 30 kg/m2)	211 (27.40%)	333 (38.59%)	340 (40.09%)	450 (49.89%)	
Household income to poverty ratio(%)					0.151
Low(< 1.35)	249 (35.27%)	234 (29.51%)	250 (31.45%)	248 (29.35%)	
Middle(≥1.35, < 3)	177	211	194	232	

	(25.07%)	(26.61%)	(24.40%)	(27.46%)	
High(≥3)	280 (39.66%)	348 (43.88%)	351 (44.15%)	365 (43.20%)	
Had at least 12 alcohol drinks/one year? (%)					<0.001
Yes	481 (61.90%)	587 (67.78%)	605 (71.01%)	672 (74.42%)	
No	234 (30.12%)	231 (26.67%)	213 (25.00%)	190 (21.04%)	
Not recorded	62 (7.98%)	48 (5.54%)	34 (3.99%)	41 (4.54%)	
Hypertension, n (%)					<0.001
Yes	214 (27.54%)	301 (34.76%)	336 (39.44%)	460 (50.94%)	
No	563 (72.46%)	565 (65.24%)	516 (60.56%)	443 (49.06%)	
High cholesterol levels,n(%)					<0.001
Yes	212 (27.28%)	339 (39.15%)	354 (41.55%)	424 (46.95%)	
No	563	520	493	475	

	(72.46%)	(60.05%)	(57.86%)	(52.60%)	
Not recorded	2 (0.26%)	7 (0.81%)	5 (0.59%)	4 (0.44%)	
Diabetes, n (%)					<0.001
Yes	677 (87.13%)	721 (83.26%)	705 (82.75%)	714 (79.07%)	
No	100 (12.87%)	145 (16.74%)	147 (17.25%)	189 (20.93%)	
Hyperlipidemia,n(%)					<0.001
Yes	640 (82.37%)	680 (78.52%)	653 (76.64%)	664 (73.53%)	
No	137 (17.63%)	186 (21.48%)	199 (23.36%)	239 (26.47%)	
Smoker(%)					<0.001
Never smoker	473 (60.88%)	504 (58.20%)	482 (56.57%)	474 (52.49%)	
Active smoker	156 (20.08%)	164 (18.94%)	158 (18.54%)	143 (15.84%)	
Former smoker	148 (19.05%)	198 (22.86%)	212 (24.88%)	286 (31.67%)	
Sleep disorder, n (%)					<0.001
Yes	49	103	85	127	

	(6.31%)	(11.89%)	(9.98%)	(14.06%)	
No	728 (93.69%)	763 (88.11%)	767 (90.02%)	776 (85.94%)	
Floss usage(%)					0.010
Never(0)	206 (26.51%)	237 (27.37%)	245 (28.76%)	312 (34.55%)	
Less use(1-2)	126 (16.22%)	137 (15.82%)	133 (15.61%)	153 (16.94%)	
Medium use(3-5)	152 (19.56%)	162 (18.71%)	171 (20.07%)	155 (17.17%)	
Frequent use(6-7)	293 (37.71%)	330 (38.11%)	303 (35.56%)	283 (31.34%)	
PERIODONTITIS					<0.001
No/mild	480 (61.78%)	509 (58.78%)	497 (58.33%)	468 (51.83%)	
Moderate/severe	297 (38.22%)	357 (41.22%)	355 (41.67%)	435 (48.17%)	

Mean ±SD: P values for continuous variables were calculated from weighted logistic regression models. For categorical variables: P values were calculated by weighted chi-square tests.

Multiple regression analysis

The correlation between sUA and periodontitis is shown in Table 2 in the Supplementary Table. sUA as a continuous variable increased the incidence of moderate/severe periodontitis, OR = 1.11, (CI: 1.06, 1.17), P<0.0001. Similar results were found after adjusting for age group and sex in Model II. In Model III, after adjusting for all covariates: age, sex, race, education, family income to poverty ratio, smoking history, drinking history, dental floss frequency, obesity, hypertension, diabetes, high cholesterol, hyperlipidemia, and sleep disorders, sUA increased the incidence of moderate to severe periodontitis with an OR of 1.10 (95%CI: 1.03, 1.16), P =0.002. This means that for each unit increase in sUA, the odds of moderate/severe periodontitis increased by 10%.

To ensure the reliability of the results, in all models, sUA was associated with periodontitis (Table 2). After converting sUA from a continuous variable to a categorical variable (quartile), using the lowest quartile Q1 as a reference, the other three groups of sUA and periodontitis were associated with OR values of 1.17, 1.16 and 1.45, respectively. The results of OR values of model II and III were consistent with the trend of OR values of unadjusted model I. Periodontitis is more likely to occur with high serum uric acid concentration.

Subgroup analyses were performed to identify possible sources of heterogeneity (Figure 2). Interaction analysis showed that sUA was more strongly associated with moderate/severe periodontitis in men than in women (OR = 1.148 (95%CI :1.067, 1.236), P=0.0002), and was also more strongly associated with moderate/severe periodontitis in non-Hispanic whites and blacks. The OR values were 1.228 and 1.212, respectively (P <

0.05).

Table 2

Association between serum uric acid (mg/dL) and periodontitis

Exposure	Adjust I	Adjust II	Adjust III
**Serum uric acid (mg/dL, mean ± SD)**	1.11 (1.06, 1.17) <0.0001	1.10 (1.04, 1.16) 0.0003	1.10 (1.03, 1.16) 0.0020
**Serum uric acid categories(%)**			
**Q1(1.1-4.3)**	1.0	1.0	1.0
**Q2(4.4-5.2)**	1.13 (0.93, 1.38) 0.2149	1.13 (0.92, 1.38) 0.2551	1.17 (0.93, 1.48) 0.1793
**Q3(5.3-6.2)**	1.15 (0.95, 1.41) 0.1567	1.11 (0.90, 1.37) 0.3205	1.16 (0.92, 1.47) 0.2137
**Q4(6.3-13.3)**	1.50 (1.24, 1.83) <0.0001	1.44 (1.17, 1.76) 0.0005	1.45 (1.14, 1.83) 0.0023
**p-value for trend**	<0.0001	0.0009	0.0039

Model 1: Unadjusted covariates.

Model 2: Adjusted for age, sex, and race/ethnicity.

Model 3: adjusted for age, sex, race/ethnicity, education, household income/poverty ratio, smoking behavior, alcohol consumption, dental floss frequency, obesity, hypertension, diabetes, high cholesterol, hyperlipidemia, and sleep disorders.

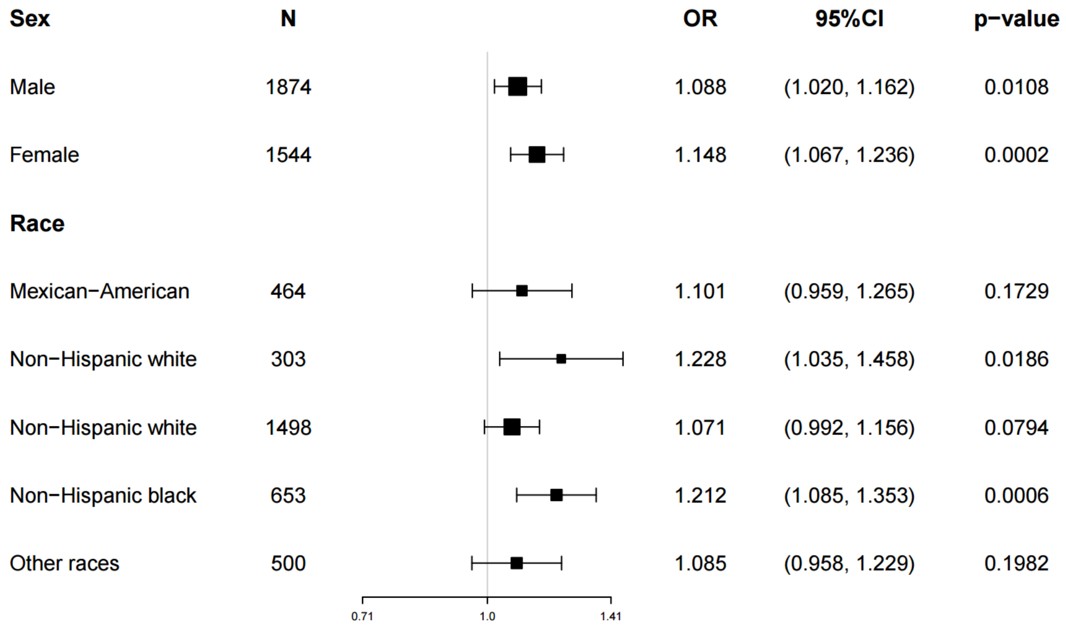

Figure 2

Subgroup analyses of the association between moderate/severe periodontitis and quartiles of serum uric acid concentration by sex and race/ethnicity (NHANES 2009-2014; n=3398).

Linear relationship exploration

After adjusting for potential confounders, curve-fitting analysis showed a linear relationship between sUA and periodontitis (FIG. 3). As can be seen from the figure, the line generally shows an upward trend, and studies have shown that serum uric acid concentration is linearly and positively related to the risk of periodontitis.

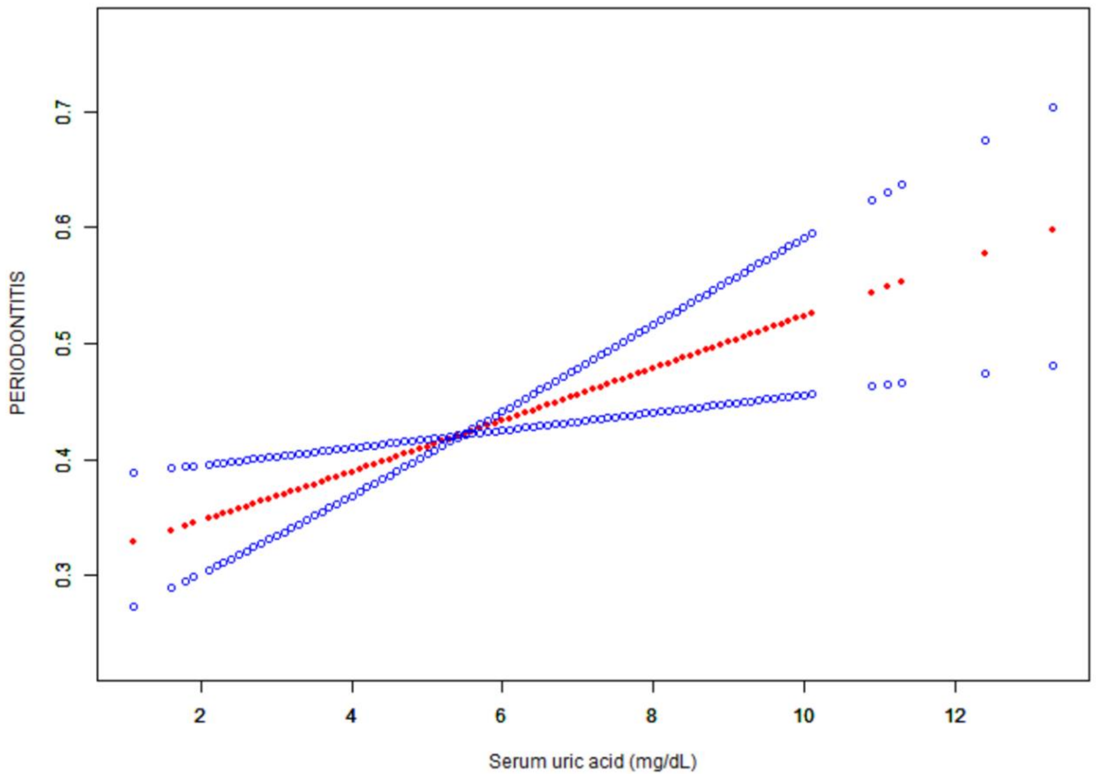

Figure 3: A linear association was found between serum uric acid concentration and periodontitis. Solid lines represent smooth curve fits between variables. The blue bands indicate 95% of the fitted confidence intervals. Adjustment for age, sex, race/ethnicity, education, household income/poverty ratio, smoking behavior, alcohol consumption, dental floss frequency, obesity, hypertension, diabetes, high cholesterol, hyperlipidemia, and sleep disorders.

In the subgroup analysis, we found that the association between serum uric acid concentration and periodontitis was different according to race/ethnicity and gender. We attempted to identify nonlinear relationships stratified by sex and race/ethnicity using generalized additive models and smooth curve fitting. Curve-fitting analysis showed a linear relationship between sUA and periodontitis in other Hispanics, non-Hispanic whites,

and other races (Figure 4,5), with a slow upward trend in Mexican Americans, whereas in non-Hispanic blacks, the association between serum uric acid and periodontitis was an inverted U-shaped curve, and a two-segment linear regression model was used to identify inflamation points, turning points: sUA 8.1mg/dL, P < 0.05. Similarly, there is a turning point for women. The incidence of periodontitis did not increase with sUA after the turning points of 4.3 mg/dL and 7.7mg/dL in women(Table 3). In summary, the association between sUA and periodontitis incidence in women and non-Hispanic blacks followed a U-shaped curve.

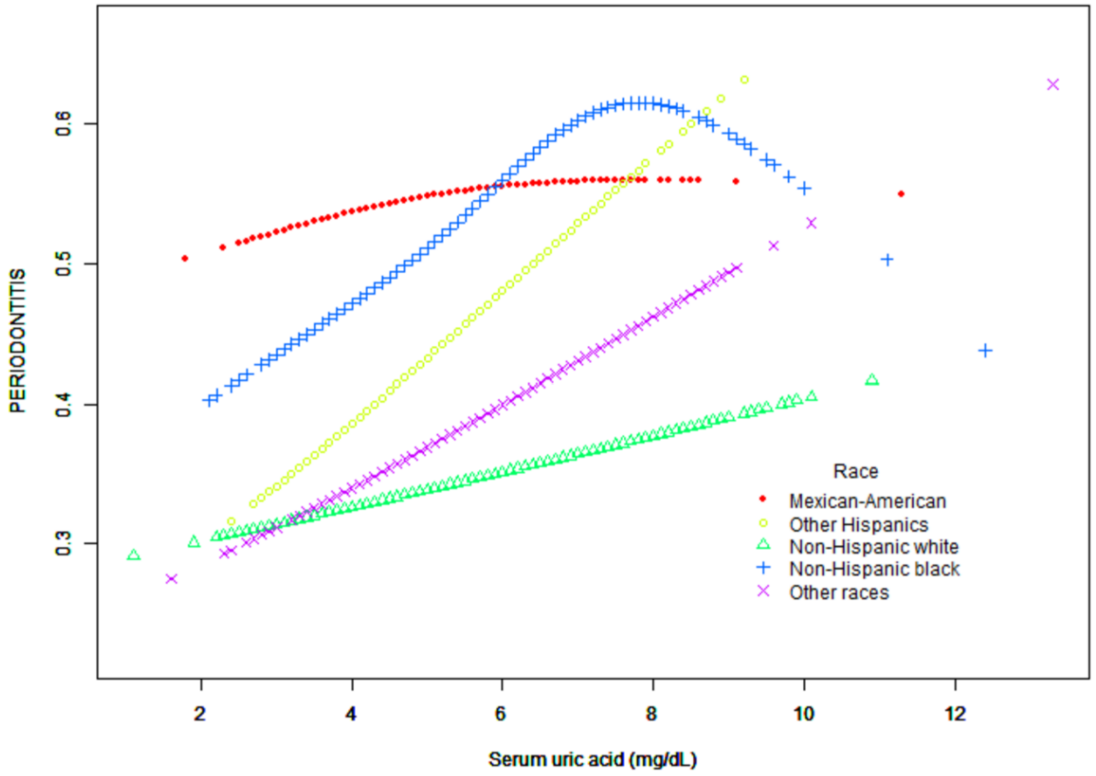

Figure 4: Associations between uric acid and periodontitis stratified by race/ethnicity.

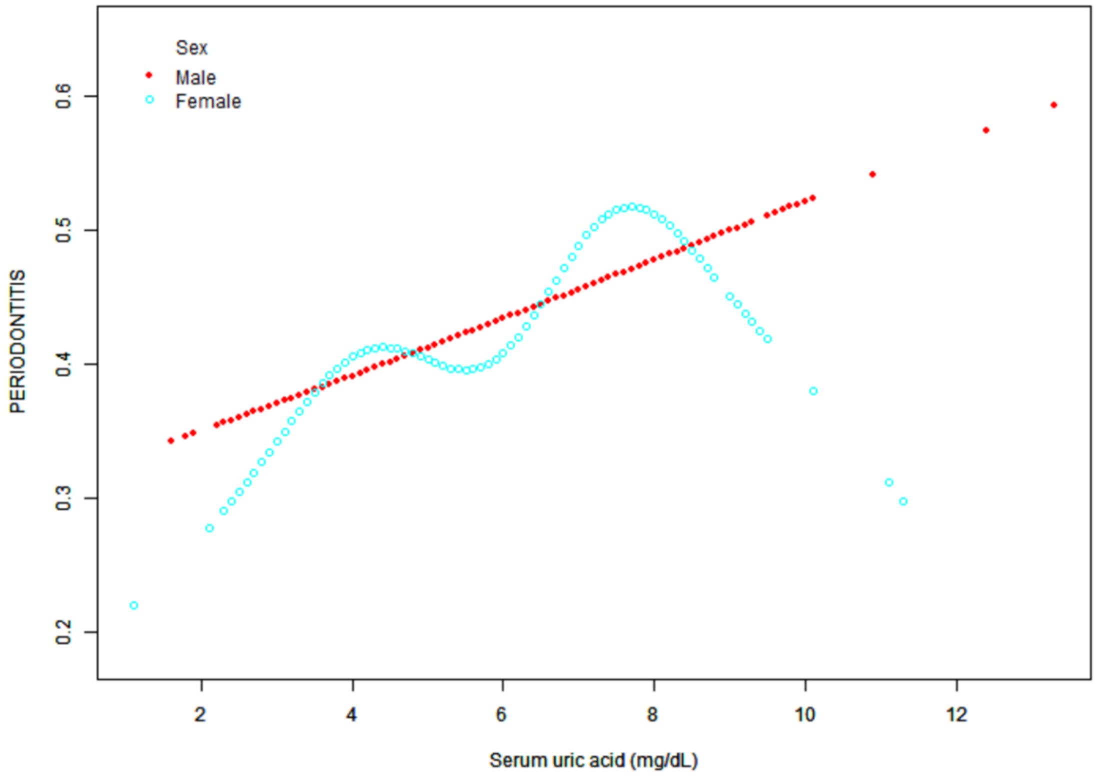

Figure 5;The association between uric acid and periodontitis was stratified by sex.

Table 3 Results of the two-segment linear regression model for the non-Hispanic black subgroup

For Exposure: Serum uric acid (mg/dL, mean ± SD)

Outcome	Non-Hispanic black	
	OR (95%CI)	P value
Fitting by weighted linear regression model	1.21 (1.01, 1.45)	0.0385
Fitting by weighted linear regression model		
Inflection point(K)	6.6	
< K	1.09 (0.89, 1.35)	0.4043
> K	5.65 (1.07, 29.96)	0.0419
Log-likelihood ratio test	0.061	

Outcome	Female		
	OR (95%CI)P value		
Fitting by weighted linear regression model	1.04 (0.88, 1.24) 0.6356	1.09 (0.97, 1.22) 0.1289	1.09 (1.00, 1.20) 0.0460

Fitting by weighted linear regression model			
Inflection point(K)	4.3	6.2	7.7
< K	1.36 (0.91, 2.04)<br>0.1338	1.01 (0.87, 1.16)<br>0.9177	1.12 (1.02, 1.24)<br>0.0214
> K	0.86 (0.62, 1.18)<br>0.3410	1.80 (1.01, 3.22)<br>0.0458	0.81 (0.48, 1.36)<br>0.4268
Log-likelihood ratio test	0.145	0.082	0.249

Discussion

The current cross-sectional survey of a nationally representative sample of US participants was designed to investigate the association of sUA with periodontitis. After adjusting for all confounding factors, the results showed that the incidence of periodontitis was statistically significantly higher with the increase of baseline sUA level, indicating that higher baseline sUA level is a risk factor for periodontitis. However, the association between sUA levels and periodontitis incidence followed a U-shaped curve for women (turning point: sUA 4.3 mg/dL, sUA 6.2 mg/dL, sUA 7.7 mg/dL,) and non-Hispanic blacks (turning point: sUA 6.6 mg/dL).

The first interesting finding was that the relationship between sUA and the incidence of periodontitis in women followed a U-shaped curve. Different from men, the incidence of periodontitis decreased with the increase of sUA after the tipping point (4.3 mg/dL) and the tipping point (7.7 mg/dL) in women. This suggests that there may be a different relationship between sUA and periodontal teeth in different gender populations. There is potential

evidence that gender is a factor worth considering in the association between sUA and the incidence of periodontitis. Sex steroids play a role in the prevalence of periodontitis[25].Firstly, it is reflected in its effect on bone metabolism. Estrogen is a key regulator of bone metabolism and inhibits the expression of important inflammatory cytokines in bone resorption, and estrogen deficiency may lead to more intense gingival inflammation during periodontitis and subsequent oral bone loss[26 27].Studies have shown that men with lower levels of bioavailable testosterone affected by sex hormone-binding globulin have a higher risk of periodontitis [25 28].The second is reflected in the function of sex steroid hormones regulating cells involved in the immune response. Females appear to have higher reactive and protective cell-mediated and humoral immune responses to antigenic challenge compared to males[29].Women with periodontitis had higher levels of antibodies to Porphyromonas gingivalis than men [30].This can be attributed to the attenuation of neutrophils in the presence of estrogen [31] , as well as the inhibition of T cell and B cell lymphopoiesis and activation of B cell function by estrogen [32].At the same time, the higher prevalence of chronic periodontitis in men reflects poor lifestyle or environmental factors, such as oral hygiene habits, higher cigarette smoking and lower use of oral health services[33].However, the lack of sex-specific results in randomized controlled trials limits the understanding of sex-specific results after interventions related to periodontitis [34].Therefore, in order to understand the mechanism behind the sex difference between sUA and periodontitis, further studies on the role of sex hormones are needed. Oral health care providers must be made aware of the gender bias contained in the evidence to take this into account in clinical decision making.

A second interesting finding was that the relationship between sUA and periodontitis incidence in non-Hispanic blacks followed an inverted U-shaped curve. When sUA levels reached 6.6 mg/dL, the incidence of periodontitis decreased. Some studies have proposed that periodontitis patients with different racial/ethnic backgrounds have different oral microbial profiles[35].Experiments have shown that black people have a stronger systemic neutrophil response to dental plaque accumulation [36].Moreover, the difference in diet between non-Hispanic blacks and whites makes the reduction of serum uric acid level less effective than that of whites [37].However, this is not consistent with our experimental results. To date, there is limited evidence linking sUA and periodontitis in different ethnic groups. We cannot explain this U-shaped curve from the existing evidence, and future prospective studies with large samples in different ethnic groups are needed to further verify it.

The relationship between UA and periodontitis is increasingly known [10 38]. The current consensus is that pathologic elevation of UA levels represents proinflammatory, oxidative, and osteoclast states [15] . Elevated levels of proinflammatory cytokines such as interleukin-1β /-6 have been observed in children with hyperuricemia [20].Cabău proposed that UA could induce epigenetic reprogramming and immune metabolism of innate immune cells to enhance inflammation, a process called training immunity [39].UA can react with peroxynitrite to form free radicals [40]. It can also enhance intracellular superoxide production by increasing the activity of nicotinamide adenine dinucleotide phosphate (NADPH) oxidase [41].This intracellular oxidative stress together with UA-induced inflammatory cytokines stimulates osteoclastic bone resorption and inhibits osteoblastic bone formation [15].Experiments have shown that increased serum UA may reduce the pH

value in the oral cavity or periodontal pocket, promote the growth and reproduction of acid-producing periodontal pathogens such as Prevotella, and increase the incidence of periodontitis[42].Therefore, sUA clearly affects systemic inflammation and leads to chronic inflammatory diseases, possibly increasing the incidence of periodontitis.

he strength of this study compared to other similar studies is that it discusses not only the baseline characteristics of participants with different sUA concentrations, but also the smooth curve fitting between sUA concentration and periodontitis. Second, the sample size was large, including 3398 participants, including a representative sample of a multiethnic population, performing further subgroup analyses for sensitivity testing, and adjusting for many potential confounders, providing strong evidence for quantitative assessment of the association between sUA concentration and periodontitis index levels.

However, this experiment has some limitations. First, the limited amount of data available in the NHANES database prevented us from being more thorough about the effect of sUA on periodontitis. Second, because our results are based on the population of the United States, they may not apply to other countries. Third, due to the cross-sectional design of this study, the causal relationship between sUA and HTN cannot be elucidated. Finally, bias due to other potential confounders that were not adjusted for in this study cannot be excluded.

Conclusion

In conclusion, this cross-sectional study suggests that sUA levels are positively associated with the occurrence of periodontitis. However, the association between sUA levels and the occurrence of periodontitis in women and non-Hispanic blacks followed a

U-shaped curve. sUA may directly or indirectly contribute to the global burden of periodontal disease, but there is little evidence for a directed association between sUA and periodontitis. This study further supports that high sUA level is a risk factor for periodontitis and may be helpful for the control of periodontitis. It also provides new insights into whether it can be used as an indicator to assess the risk or progression of periodontitis. More studies are needed to confirm the relationship between sUA and periodontitis.

Declarations:

Availability of data and materials

Not applicable.

Authors' contributions：

Bai Jingjing designed the research, contributed to data interpretation,and drafted the manuscript.Zhou Chenying,Liu Ye,Ding Ming and Zhang Zhonghua,contributed to data interpretation and the manuscript. Chen Zhu, Feng Ping and Song Jukun reviewed the article. All authors read and approved the final manuscript.

Ethics Approval and Consent to Participate：

Ethical approval and review were not sought or obtained for this study given that it was secondary analysis of publicly available, de-identified data.

Funding:

This study was partially supported by the Science and Technology Foundation of Guizhou Provincial Health Commission (GZWKJ2023-063).

Conflict of Interests:

The authors declare that the research was conducted in the absence of any commercial or financial relationships that could be construed as a potential conflict of interest.

References

[1]. Fischer RG, Lira Junior R, Retamal-Valdes B, et al. Periodontal disease and its impact on general health in Latin America. Section V: Treatment of periodontitis. *Braz Oral Res* 2020;34(supp1 1):e026. doi: 10.1590/1807-3107bor-2020.vol34.0026 [published Online First: 20200409]

[2]. Trindade D, Carvalho R, Machado V, et al. Prevalence of periodontitis in dentate people between 2011 and 2020: A systematic review and meta-analysis of epidemiological studies. *J Clin Periodontol* 2023;50(5):604-626. doi: 10.1111/jcpe.13769 [published Online First: 20230120]

[3]. Norhammar A, Kjellström B, Habib N, et al. Undetected Dysglycemia Is an Important Risk Factor for Two Common Diseases, Myocardial Infarction and Periodontitis: A Report From the PAROKRANK Study. *Diabetes Care* 2019;42(8):1504-1511. doi:

10.2337/dc19-0018 [published Online First: 20190610]

[4]. Schwahn C, Frenzel S, Holtfreter B, et al. Effect of periodontal treatment on preclinical Alzheimer's disease-Results of a trial emulation approach. *Alzheimers Dement* 2022;18(1):127-141. doi: 10.1002/alz.12378 [published Online First: 20210529]

[5]. Priyamvara A, Dey AK, Bandyopadhyay D, et al. Periodontal Inflammation and the Risk of Cardiovascular Disease. *Curr Atheroscler Rep* 2020;22(7):28. doi: 10.1007/s11883-020-00848-6 [published Online First: 20200608]

[6]. Chen MX, Zhong YJ, Dong QQ, et al. Global, regional, and national burden of severe periodontitis, 1990-2019: An analysis of the Global Burden of Disease Study 2019. *J Clin Periodontol* 2021;48(9):1165-1188. doi: 10.1111/jcpe.13506 [published Online First: 20210707]

[7]. Hajishengallis G. Immunomicrobial pathogenesis of periodontitis: keystones, pathobionts, and host response. *Trends Immunol* 2014;35(1):3-11. doi: 10.1016/j.it.2013.09.001 [published Online First: 20131023]

[8]. Kinane DF, Stathopoulou PG, Papapanou PN. Periodontal diseases. *Nat Rev Dis Primers* 2017;3:17038. doi: 10.1038/nrdp.2017.38 [published Online First: 20170622]

[9]. Wang Y, Andrukhov O, Rausch-Fan X. Oxidative Stress and Antioxidant System in Periodontitis. *Front Physiol* 2017;8:910. doi: 10.3389/fphys.2017.00910 [published Online First: 20171113]

[10]. Chen ZY, Ye LW, Zhao L, et al. Hyperuricemia as a potential plausible risk factor for periodontitis. *Med Hypotheses* 2020;137:109591. doi: 10.1016/j.mehy.2020.109591 [published Online First: 20200123]

[11]. El Ridi R, Tallima H. Physiological functions and pathogenic potential of uric acid: A review. *J Adv Res* 2017;8(5):487-493. doi: 10.1016/j.jare.2017.03.003 [published Online First: 20170314]

[12]. Yanai H, Adachi H, Hakoshima M, et al. Molecular Biological and Clinical Understanding of the Pathophysiology and Treatments of Hyperuricemia and Its Association with Metabolic Syndrome, Cardiovascular Diseases and Chronic Kidney Disease. *Int J Mol Sci* 2021;22(17) doi: 10.3390/ijms22179221 [published Online First: 20210826]

[13]. Cortese F, Giordano P, Scicchitano P, et al. Uric acid: from a biological advantage to a potential danger. A focus on cardiovascular effects. *Vascul Pharmacol* 2019;120:106565. doi: 10.1016/j.vph.2019.106565 [published Online First: 20190529]

[14]. Crawley WT, Jungels CG, Stenmark KR, et al. U-shaped association of uric acid to overall-cause mortality and its impact on clinical management of hyperuricemia. *Redox Biol* 2022;51:102271. doi: 10.1016/j.redox.2022.102271 [published Online First: 20220217]

[15]. Lin KM, Lu CL, Hung KC, et al. The Paradoxical Role of Uric Acid in Osteoporosis. *Nutrients* 2019;11(9) doi: 10.3390/nu11092111 [published Online First: 20190905]

[16]. Zhou J, Hu H, Huang R. A pilot study of the metabolomic profiles of saliva from female orthodontic patients with external apical root resorption. *Clin Chim Acta* 2018;478:188-193. doi: 10.1016/j.cca.2017.12.046 [published Online First: 20171229]

[17]. Banu S, Jabir NR, Mohan R, et al. Correlation of Toll-like receptor 4, interleukin-18, transaminases, and uric acid in patients with chronic periodontitis and healthy adults. *J Periodontol* 2015;86(3):431-439. doi: 10.1902/jop.2014.140414 [published Online First: 20141027]

[18]. Tsai KZ, Su FY, Cheng WC, et al. Associations between metabolic biomarkers and localized stage II/III periodontitis in young adults: The CHIEF Oral Health study. *J Clin Periodontol* 2021;48(12):1549-1558. doi: 10.1111/jcpe.13555 [published Online First: 20211006]

[19]. Sato K, Yamazaki K, Kato T, et al. Obesity-Related Gut Microbiota Aggravates Alveolar Bone Destruction in Experimental Periodontitis through Elevation of Uric Acid. *mBio* 2021;12(3):e0077121. doi: 10.1128/mBio.00771-21 [published Online First: 20210601]

[20]. Di Y, Wang J, Chen Y, et al. Elevated Interleukin 1β and Interleukin 6 Levels in the Serum of Children With Hyperuricemia. *J Clin Rheumatol* 2018;24(2):65-69. doi: 10.1097/rhu.0000000000000611

[21]. Veronese N, Carraro S, Bano G, et al. Hyperuricemia protects against low bone mineral density, osteoporosis and fractures: a systematic review and meta-analysis. *Eur J Clin Invest* 2016;46(11):920-930. doi: 10.1111/eci.12677 [published Online First: 20161005]

[22]. Curtin LR, Mohadjer LK, Dohrmann SM, et al. The National Health and Nutrition Examination Survey: Sample Design, 1999-2006. *Vital Health Stat 2* 2012(155):1-39.

[23]. Eke PI, Dye BA, Wei L, et al. Update on Prevalence of Periodontitis in Adults in the United States: NHANES 2009 to 2012. *J Periodontol* 2015;86(5):611-622. doi: 10.1902/jop.2015.140520 [published Online First: 20150217]

[24]. Tran DT, Gay I, Du XL, et al. Assessment of partial-mouth periodontal examination protocols for periodontitis surveillance. *J Clin Periodontol* 2014;41(9):846-852. doi: 10.1111/jcpe.12285 [published Online First: 20140715]

[25]. Steffens JP, Wang X, Starr JR, et al. Associations Between Sex Hormone Levels and

Periodontitis in Men: Results From NHANES III. *J Periodontol* 2015;86(10):1116-1125. doi: 10.1902/jop.2015.140530 [published Online First: 20150611]

[26]. Lerner UH. Inflammation-induced bone remodeling in periodontal disease and the influence of post-menopausal osteoporosis. *J Dent Res* 2006;85(7):596-607. doi: 10.1177/154405910608500704

[27]. Khosla S, Monroe DG. Regulation of Bone Metabolism by Sex Steroids. *Cold Spring Harb Perspect Med* 2018;8(1) doi: 10.1101/cshperspect.a031211 [published Online First: 20180102]

[28]. Su X, Jin K, Zhou X, et al. The association between sex hormones and periodontitis among American adults: A cross-sectional study. *Front Endocrinol (Lausanne)* 2023;14:1125819. doi: 10.3389/fendo.2023.1125819 [published Online First: 20230214]

[29]. Taneja V. Sex Hormones Determine Immune Response. *Front Immunol* 2018;9:1931. doi: 10.3389/fimmu.2018.01931 [published Online First: 20180827]

[30]. Ebersole JL, Al-Sabbagh M, Gonzalez OA, et al. Ageing effects on humoral immune responses in chronic periodontitis. *J Clin Periodontol* 2018;45(6):680-692. doi: 10.1111/jcpe.12881 [published Online First: 20180426]

[31]. Miyagi M, Aoyama H, Morishita M, et al. Effects of sex hormones on chemotaxis of human peripheral polymorphonuclear leukocytes and monocytes. *J Periodontol* 1992;63(1):28-32. doi: 10.1902/jop.1992.63.1.28

[32]. Moulton VR. Sex Hormones in Acquired Immunity and Autoimmune Disease. *Front Immunol* 2018;9:2279. doi: 10.3389/fimmu.2018.02279 [published Online First:

20181004]

[33]. Haytac MC, Ozcelik O, Mariotti A. Periodontal disease in men. *Periodontol 2000* 2013;61(1):252-265. doi: 10.1111/j.1600-0757.2011.00410.x

[34]. Michelson C, Al-Abedalla K, Wagner J, et al. Lack of attention to sex and gender in periodontitis-related randomized clinical trials: A meta-research study. *J Clin Periodontol* 2022;49(12):1320-1333. doi: 10.1111/jcpe.13707 [published Online First: 20220904]

[35]. Wang BY, Lu T, Cai Q, et al. Potential Microbiological Risk Factors Associated With Periodontitis and Periodontal Health Disparities. *Front Cell Infect Microbiol* 2021;11:789919. doi: 10.3389/fcimb.2021.789919 [published Online First: 20211117]

[36]. Wahaidi VY, Dowsett SA, Eckert GJ, et al. Neutrophil response to dental plaque by gender and race. *J Dent Res* 2009;88(8):709-714. doi: 10.1177/0022034509339019

[37]. McCormick N, Lu N, Yokose C, et al. Racial and Sex Disparities in Gout Prevalence Among US Adults. *JAMA Netw Open* 2022;5(8):e2226804. doi: 10.1001/jamanetworkopen.2022.26804 [published Online First: 20220801]

[38]. Hou W, Xia X, Li Y, et al. Recent progress and perspectives on the relationship between hyperuricemia and periodontitis. *Front Immunol* 2022;13:995582. doi: 10.3389/fimmu.2022.995582 [published Online First: 20221116]

[39]. Cabău G, Crișan TO, Klück V, et al. Urate-induced immune programming: Consequences for gouty arthritis and hyperuricemia. *Immunol Rev* 2020;294(1):92-105. doi: 10.1111/imr.12833 [published Online First: 20191219]

[40]. Santos CX, Anjos EI, Augusto O. Uric acid oxidation by peroxynitrite: multiple reactions,

free radical formation, and amplification of lipid oxidation. *Arch Biochem Biophys* 1999;372(2):285-294. doi: 10.1006/abbi.1999.1491

[41]. Sautin YY, Johnson RJ. Uric acid: the oxidant-antioxidant paradox. *Nucleosides Nucleotides Nucleic Acids* 2008;27(6):608-619. doi: 10.1080/15257770802138558

[42]. Zhao J, Huang Y. Salivary uric acid as a noninvasive biomarker for monitoring the efficacy of urate-lowering therapy in a patient with chronic gouty arthropathy. *Clin Chim Acta* 2015;450:115-120. doi: 10.1016/j.cca.2015.08.005 [published Online First: 20150811]

Table 1

Weighted characteristics of the study population based on quartiles of serum uric acid.

Table 2

Association between serum uric acid (mg/dL) and periodontitis

Table 3

Results of the two-segment linear regression model for the non-Hispanic black subgroup

Figure 1

Sample screening flow chart.

Figure 2

Subgroup analyses of the association between moderate/severe periodontitis and quartiles

of serum uric acid concentration by sex and race/ethnicity (NHANES 2009-2014; n=3398).

Figure 3

A linear association was found between serum uric acid concentration and periodontitis. Solid lines represent smooth curve fits between variables. The blue bands indicate 95% of the fitted confidence intervals. Adjustment for age, sex, race/ethnicity, education, household income/poverty ratio, smoking behavior, alcohol consumption, dental floss frequency, obesity, hypertension, diabetes, high cholesterol, hyperlipidemia, and sleep disorders.

Figure 4

Associations between uric acid and periodontitis stratified by race/ethnicity.

Figure 5

The association between uric acid and periodontitis was stratified by sex.

Figure Click here to access/download;Figure;Figure 1.jpg

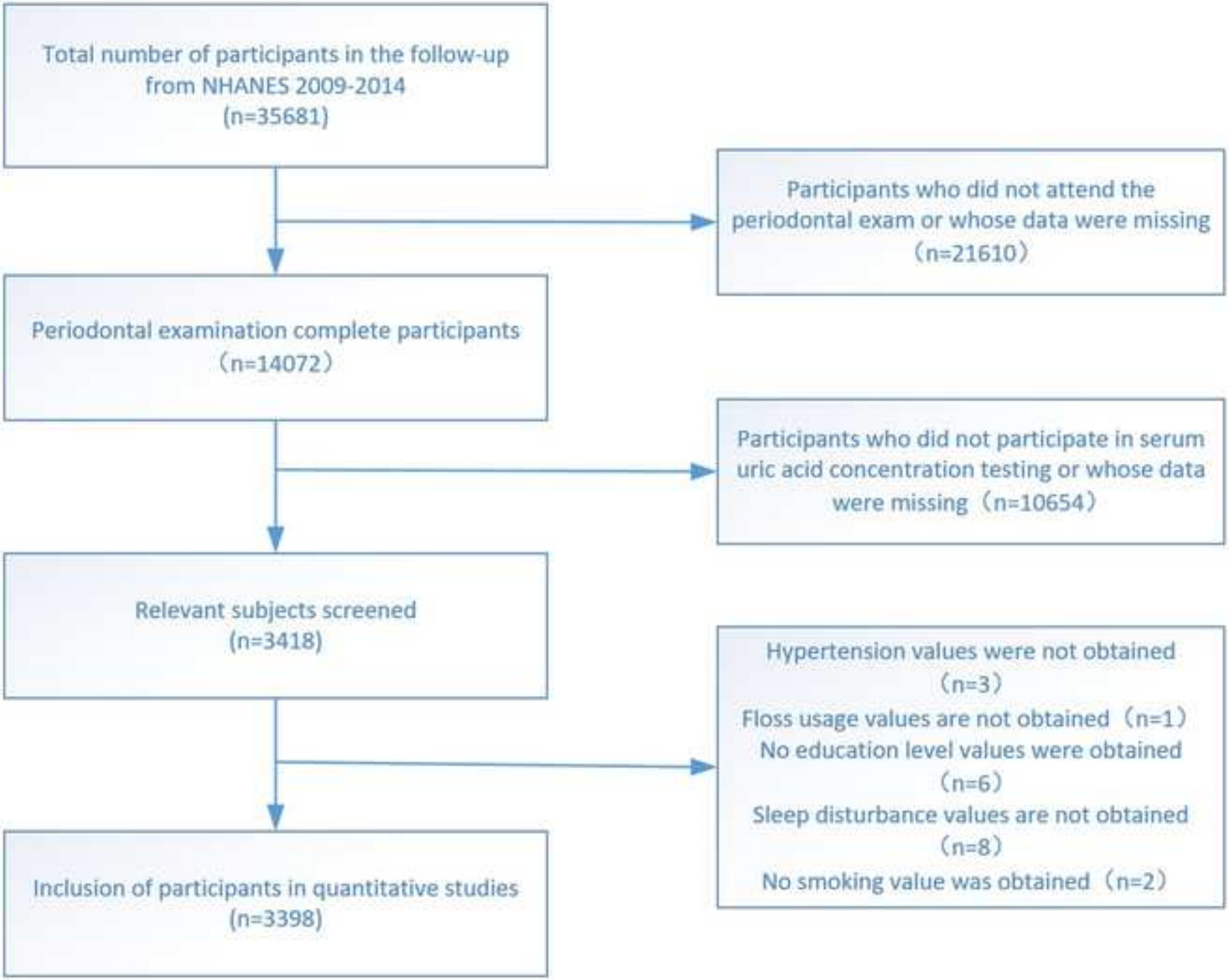

Figure

Click here to access/download;Figure;Figure 2.jpg

Sex	N	OR	95%CI	p−value
Male	1874	1.088	(1.020, 1.162)	0.0108
Female	1544	1.148	(1.067, 1.236)	0.0002
**Race**				
Mexican−American	464	1.101	(0.959, 1.265)	0.1729
Non−Hispanic white	303	1.228	(1.035, 1.458)	0.0186
Non−Hispanic white	1498	1.071	(0.992, 1.156)	0.0794
Non−Hispanic black	653	1.212	(1.085, 1.353)	0.0006
Other races	500	1.085	(0.958, 1.229)	0.1982

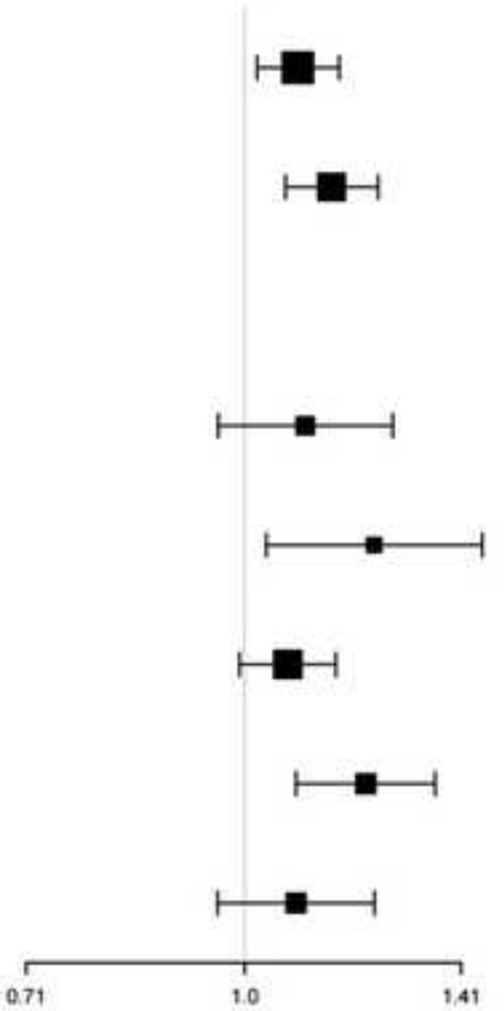

Click here to access/download;Figure;Figure 3.jpg

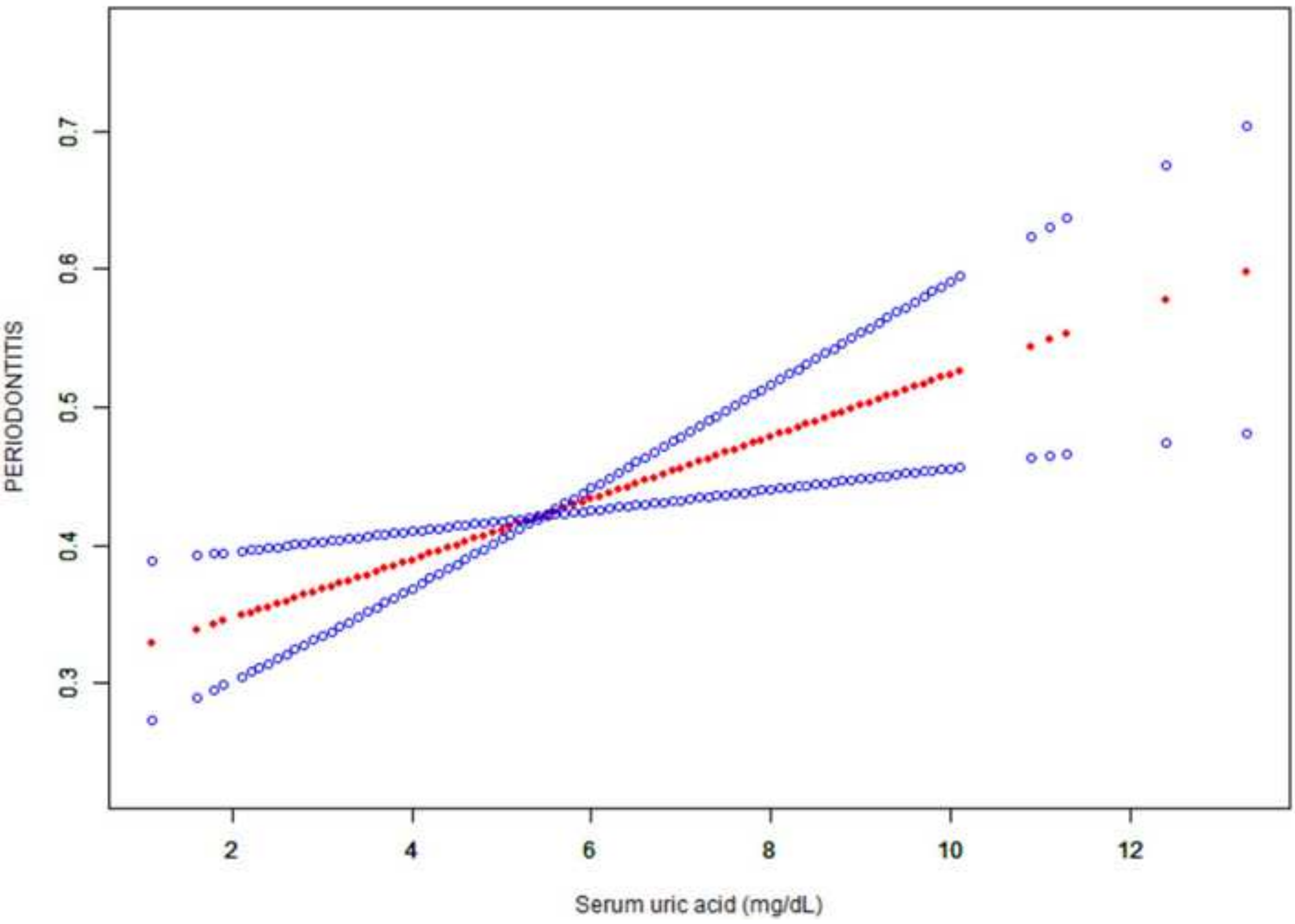

Figure

Click here to access/download;Figure;Figure 4.jpg

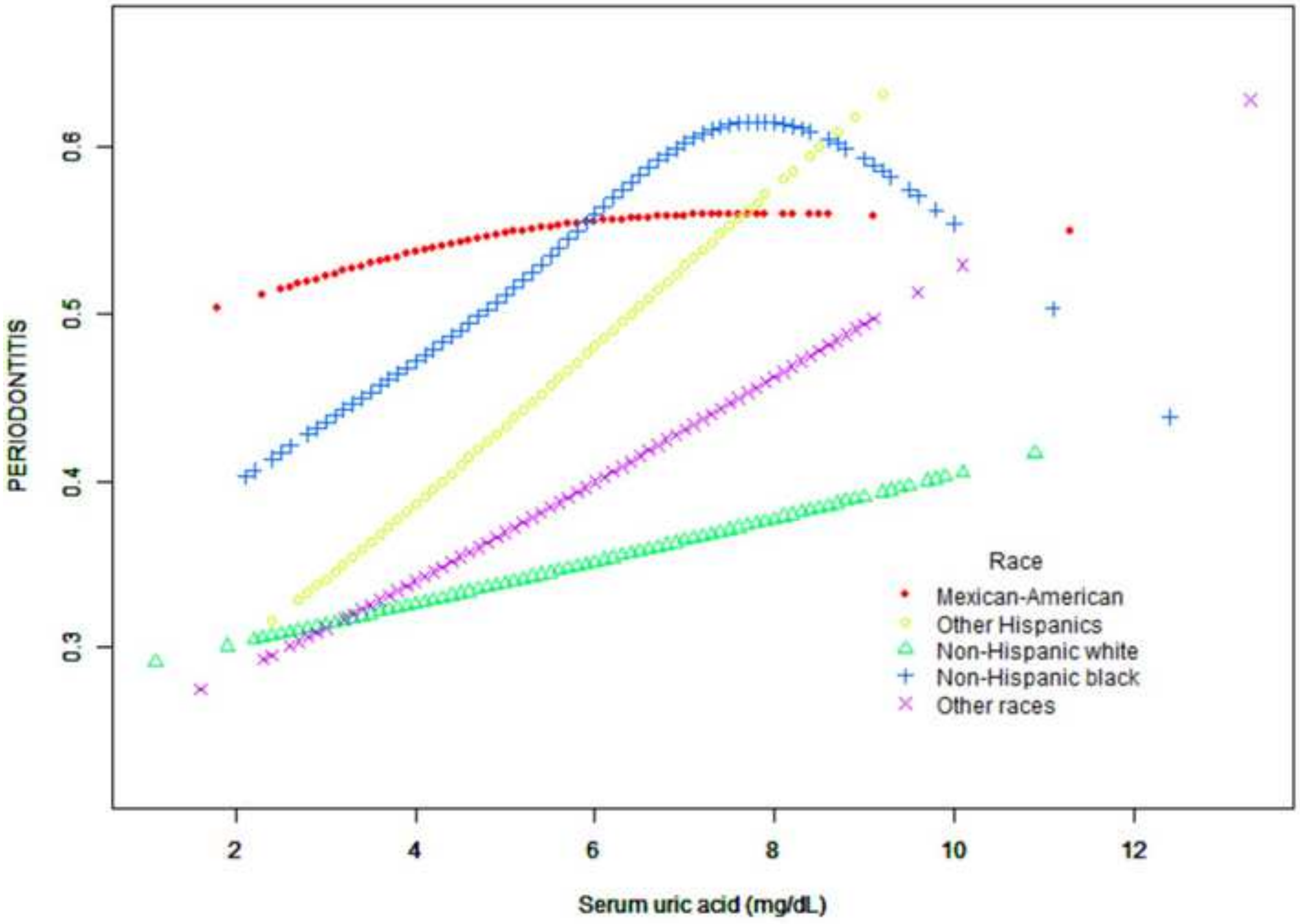

Figure

Click here to access/download;Figure;Figure 5.jpg

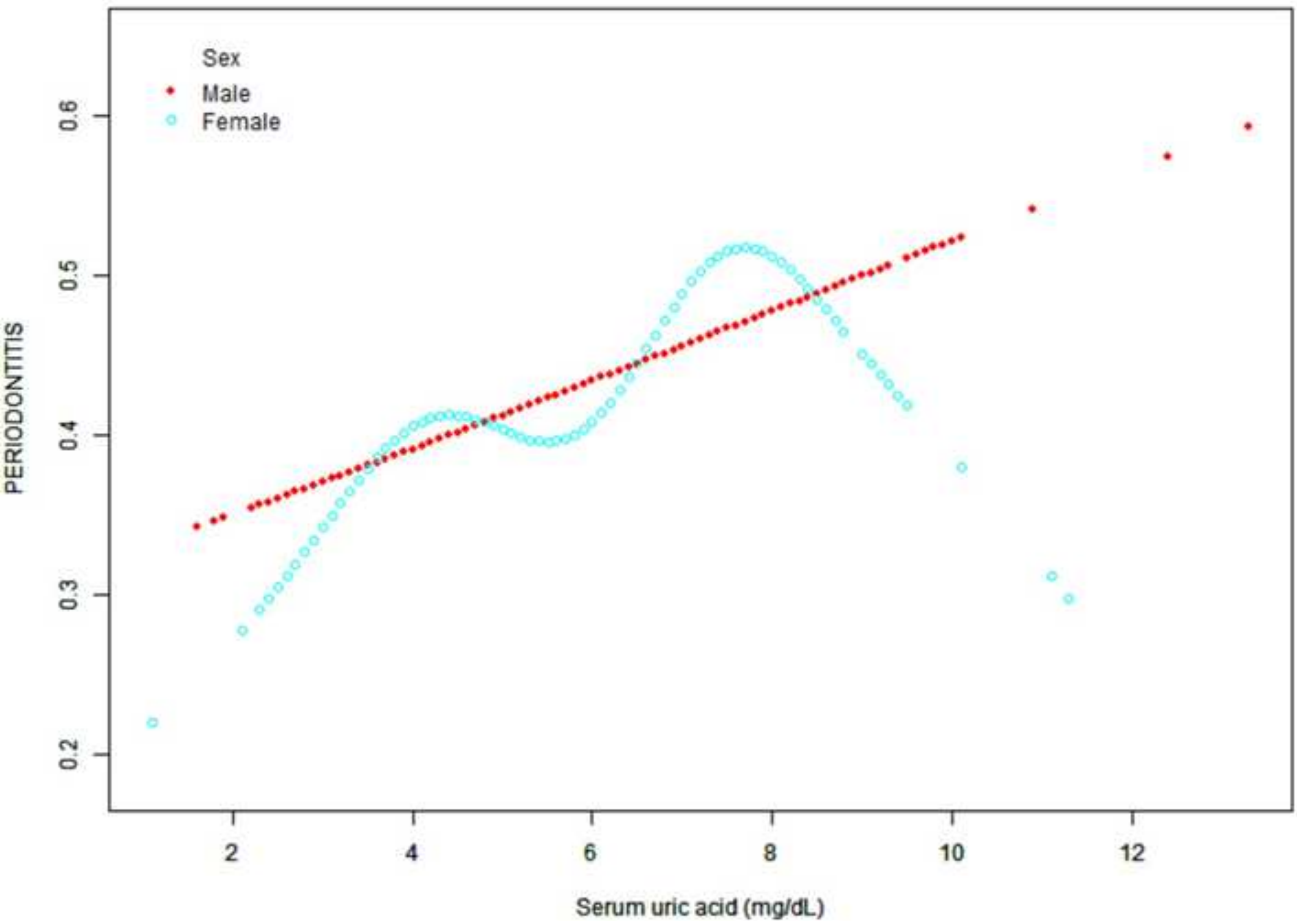

Supplement: Attachment — Submitted filename: PONE-D-24-03627.pdf [file pone.0310243.s003.pdf]
