# Supplementary material for: Relationship between serum uric acid levels and periodontitis—A cross-sectional study
Source: PLoS One. 2024 Sep 27;19(9):e0310243. doi: 10.1371/journal.pone.0310243 (PMC11432880; doi:10.1371/journal.pone.0310243)
Supplement: Attachment — Submitted filename: Opinion_PONE_26_05_2024.docx [file pone.0310243.s002.docx]

**Dear Editor Dr. Costa:**

I read with great interest the article entitled **Relationship between serum uric acid levels and periodontitis.** The topic is relevant to periodontics and the health sector.

After a careful analysis, I concluded that the article in its current form is not suitable for publication and requires a detailed review by the authors on many items, which need to be clarified for better understanding by the reader.

Therefore, I am attaching the PDF file of the article highlighted in yellow and comments on some items that I consider important and are not clear throughout the text.

I hope I have contributed to the evaluation of the article and thank you for your trust.

**Isaac**

**+55 75 99972 4972**

**Dear authors:**

I read with great interest the article entitled **Relationship between serum uric acid levels and periodontitis.** The topic is relevant to periodontics and the health sector.

After a careful analysis, I concluded that the article in its current form is not suitable for publication and requires a detailed review by the authors on many items, which need to be clarified for better understanding by the reader. The study design is cross-sectional, and many terms used and statements are not suitable for a study with this type of design. Furthermore, details need to be incorporated into the text for important items and to improve understanding of the study.

Therefore, I am attaching the PDF file of the article highlighted in yellow and comments on some items that I consider important and are not clear throughout the text. Do this to improve the article.
