# Supplementary material for: Relationship between serum uric acid levels and periodontitis—A cross-sectional study
Source: PLoS One. 2024 Sep 27;19(9):e0310243. doi: 10.1371/journal.pone.0310243 (PMC11432880; doi:10.1371/journal.pone.0310243)
Supplement: Attachment — Submitted filename: Rebuttal letter to the Academic Editor.docx [file pone.0310243.s004.docx]

1、Please ensure that your manuscript meets PLOS ONE's style requirements, including those for file naming.

Response: Thanks for your advice! I have modified the format as required.

2、We noticed you have some minor occurrence of overlapping text with the following previous publication(s), which needs to be addressed.

Response: Thank the editor for giving me the suggestion, I have finished the revision. It is your professional questions that make my article more complete.

3、We note that the grant information you provided in the ‘Funding Information’ and ‘Financial Disclosure’ sections do not match.

Response: Thank you for your question. I have modified it when I submitted it again.

4、ORCID iD

Response:I have linked the ORCID iD to the Editor Manager account as requested.

5、minimal data set

Response: Thank you for reminding me, I couldn't agree more. For the minimal data set in my article (including my graphs, tables, and data), I have uploaded the Supporting information file.

6、Please remove your figures.

Response: I have removed the figures from the manuscript as requested and uploaded them as files, named "S1_Fig", "S2_Fig", "S3_Fig", "S4_Fig" and "S5_Fig".

7、Authors should pay attention to the appropriate terminology for a cross-sectional study, especially regarding its temporality.

Response: Thank you for your professional advice. My article is a cross-sectional study, and I acknowledge that this type of study can only demonstrate an association between serum uric acid and periodontitis, without establishing a causal relationship. I have revised the manuscript to address any ambiguities related to this issue.

The hypothesis proposed in my manuscript, that "uric acid is a risk factor for periodontitis," is grounded in previous research:

Evidence 1: Uric acid has its own mechanism of enhancing inflammation.

Evidence 2: Elevated uric acid levels may reduce the pH value in the oral cavity or periodontal pocket, promoting the growth and reproduction of acid-producing periodontal pathogens such as Prevotella, thereby increasing the prevalence of periodontitis.

Evidence 3: Some studies have observed improvement in periodontitis following the control of uric acid levels.

(More details are available in the manuscript.)
